# Clinical Significance of Serum Hemeoxygenase-1 as a New Biomarker for the Patients with Interstitial Pneumonia

**DOI:** 10.1155/2018/7260178

**Published:** 2018-11-22

**Authors:** Kota Murohashi, Yu Hara, Kanako Shinada, Kenjiro Nagai, Masaharu Shinkai, Akihiko Kawana, Takeshi Kaneko

**Affiliations:** ^1^Department of Pulmonology, Yokohama City University Graduate School of Medicine, Yokohama, Japan; ^2^Department of Pulmonology, Tokyo-Shinagawa Hospital, Shinagawa, Japan; ^3^Division of Infectious Diseases and Pulmonary Medicine, Department of Internal Medicine, National Defense Medical College, Saitama, Japan

## Abstract

**Background:**

Serum hemeoxygenase-1 (HO-1) has been proposed to be a biomarker of lung disease activity and prognosis. The present study aimed at evaluating whether HO-1 could be a useful marker for evaluating disease activity and predicting prognosis in patients with interstitial pneumonia (IP).

**Materials and Methods:**

Serum HO-1 levels of newly diagnosed or untreated patients with IP were measured at hospitalization. We evaluated the relationships between serum HO-1 and other serum biomarkers, high resolution CT (HRCT) findings, and hospital mortality.

**Results:**

Twenty-eight patients with IP, including 14 having an acute exacerbation (AE) and 14 not having an AE, were evaluated. The patients having an AE had significantly higher HO-1 levels than those not having an AE (53.5 ng/mL vs. 24.1 ng/mL; *p* < 0.001), and the best cut-off level to discriminate between having an AE or not having an AE was 41.6 ng/mL. Serum HO-1 levels were positively correlated with serum levels of surfactant protein-D (*r*=0.66, *p* < 0.001) and the ground glass opacity score (calculated from HRCT; *r*=0.40, *p*=0.036). Patients who subsequently died in hospital had presented with significantly higher HO-1 levels than those who did not die in hospital (64.8 ng/mL vs. 32.0 ng/mL; *p*=0.009).

**Conclusion:**

Serum HO-1 may serve as a useful biomarker for detecting AE or predicting hospital mortality in patients with IP.

## 1. Introduction

Hemeoxygenase-1 (HO-1) is a 32 kDa heat shock protein that converts heme to carbon monoxide, iron, and bilirubin [1, 2]. HO-1 is released from alveolar macrophages, bronchial epithelial cells, and inflammatory cells upon exposure to various stimuli, including cytokines, hyperoxia and hypoxia, exogenous nitric oxide, and diesel exhaust particles [3]. HO-1 has been reported to be upregulated in the lungs of patients with interstitial lung disease (ILD), including silicosis, sarcoidosis, standard interstitial pneumonia (IP), and acute fibrinous and organizing pneumonia [4–7]. Additionally, serum HO-1 levels in the peripheral blood are reported to be elevated in patients with silicosis and in patients with acute respiratory distress syndrome (ARDS) [8–11]. However, the clinical significance of serum HO-1 in patients IP is unknown. In the present work, we measured serum HO-1 levels in patients with newly diagnosed and untreated IP and evaluated the correlations between serum HO-1 and other established parameters, including clinical outcome.

## 2. Materials and Methods

### 2.1. Study Patients and the Diagnosis of IP

This study enrolled a total of 28 newly diagnosed and untreated IP patients who, from April 2011 to March 2018, had been admitted to the hospital and were able to provide informed consent for participation in this research. The extracted data included the patients' medical histories, the physical examination findings, the results of blood biomarkers including an arterial blood gas analysis, and the high-resolution CT (HRCT) findings. The diagnosis of idiopathic pulmonary fibrosis (IPF) was based on the IPF consensus classification [12]. In the patients with non-IPF, the diagnosis of connective tissue disease-associated ILD (CT-ILD) was confirmed by physical findings, serological testing, and HRCT findings that were consistent with IP. Histological evaluation of lung biopsy specimens was undertaken for the exclusion of other specific diseases. The diagnosis of idiopathic nonspecific interstitial pneumonia (iNSIP) was based on established criteria [13]. All of the enrolled patients were categorized into either of two groups: those with IP not having an acute exacerbation (AE), and those with IP having an AE. For this purpose, AE was defined as an unexplained worsening of dyspnea, hypoxemia, or the worsening or severe impairment of gas exchange; new alveolar infiltration on a radiograph; and the absence of an alternative explanation such as infection, pulmonary embolism, pneumothorax, or heart failure [14–16].

### 2.2. Serum HO-1 and Other Blood Biomarker Measurements

Blood samples were obtained at hospitalization from each patient. We measured serum HO-1 along with lactate dehydrogenase (LDH; normal <225 U/L), surfactant protein- (SP-) A (SP-A; normal <43.8 ng/mL), SP-D (normal <110 ng/mL), KL-6 (normal <500 U/mL), and partial pressure of oxygen in arterial blood (PaO_2_)/a fraction of the inspiratory oxygen (FiO_2_) (PaO_2_/FiO_2_ ratio). Serum HO-1 levels were measured using the ImmunoSet™ HO-1 ELISA development set (Enzo, Farmingdale, NY, USA), according to the manufacturers' instructions. The details of this ELISA method have been described previously [11]. The control subjects for serum HO-1 levels included 28 healthy, nonsmoking adults who had been admitted to the hospital for a medical check.

### 2.3. HRCT Scoring

The HRCT findings were evaluated using the semiquantitative scoring method described by Ooi et al. [17]. The lungs were divided into six distinct zones, three on each side. Ground glass opacity (GGO) and honeycombing in the HRCT then were scored based on the percentage of disease extent in each of the 6 lung lobes. A global score was calculated by adding the scores for each abnormality in all lobes. HRCT was performed at hospitalization; each scan was independently assessed by two pulmonologists and two radiologists.

### 2.4. Statistical Analysis

All data are expressed as the median with 25^th^ to 75^th^ percentiles unless otherwise indicated. Statistical analysis was performed using JMP11 (SAS Institute, Inc., North Carolina, USA). Group comparisons were made using Wilcoxon's rank-sum test or the chi-squared test. We performed a receiver operating characteristics (ROC) curve analysis to determine the most suitable cut-off levels of serum HO-1 and other blood biomarkers for detecting an AE and predicting hospital death among patients having an AE. Nonparametric Spearman's rank correlation coefficients were calculated to assess the correlations between the serum HO-1 levels and other clinical parameters. *p* < 0.05 was considered significant.

### 2.5. Study Approval

All participants provided informed consent prior to participation in this research. All aspects of the study were approved by the Institutional Review Board of Yokohama City University Medical Center (approval number D1303019), National Defense Medical College (approval number 909), and Yokohama City University Graduate School of Medicine (approval number B170900025).

## 3. Results

### 3.1. Patient Characteristics

Clinical characteristics of patients with IP are summarized in Table 1. Twenty-eight patients with IP, including 14 having an AE and 14 not having an AE, were evaluated. The AE group included 10 IPF patients and 4 non-IPF patients. The non-AE group included 6 IPF patients and 8 non-IPF patients. The diagnoses of the non-IPF patients included of 7 cases of CT-ILD and 5 cases of iNSIP. The patients having an AE had significantly higher serum HO-1 and LDH levels, PaO_2_/FiO_2_ ratios, and GGO scores calculated from HRCT than those not having an AE.

### 3.2. Serum HO-1 Levels in IP Patients and Control Subjects

**Figure 1:**
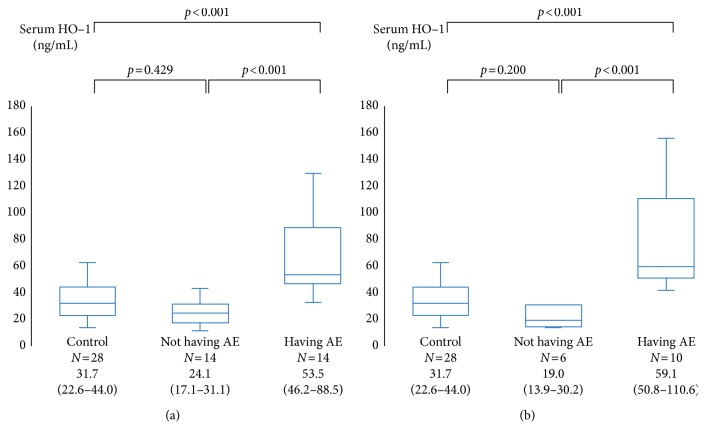
Serum hemeoxygenase-1 (HO-1) levels in interstitial pneumonia (IP) patients and control subjects. The HO-1 levels in IP patients having an acute exacerbation (AE) were significantly higher than those in IP patients not having an AE and those in control subjects (a). Also, the HO-1 levels in patients with IPF were significantly higher than those in patients not having an AE and those in control subjects (b). The center bold line is the median value; the bottom and top of the boxes represent the 25^th^ to 75^th^ percentiles, respectively; and the whiskers are 95% confidence intervals.

The median serum HO-1 level in IP patients having an AE was 53.5 ng/mL; this value was significantly higher than those in IP patients not having an AE (24.1 ng/mL, *p* < 0.001) and in control subjects (31.7 ng/mL, *p* < 0.001) (Figure 1(a)). Among patients with IPF, the median serum HO-1 level in patients having an AE was 59.1 ng/mL; this value was significantly higher than those in patients not having an AE (19.0 ng/mL, *p* < 0.001) and in control subjects (31.7 ng/mL, *p* < 0.001) (Figure 1(b)).

### 3.3. ROC Curve for Serum HO-1 Level (to Detect an AE)

The ROC curve analysis for the serum HO-1 level was evaluated to discriminate the patients having an AE from the patients not having an AE. The area under the ROC curve (AUC) was 0.91, and the best cut-off level was 41.6 ng/mL. Using this cut-off level, serum HO-1 had a sensitivity of 92% and a specificity of 82% for detecting an AE. An AUC for HO-1 was higher than that obtained for other blood biomarkers and HRCT scores (Table 2).

### 3.4. Relationship between the Serum HO-1 Level and the Blood Biomarker Level and HRCT Scores

**Figure 2:**
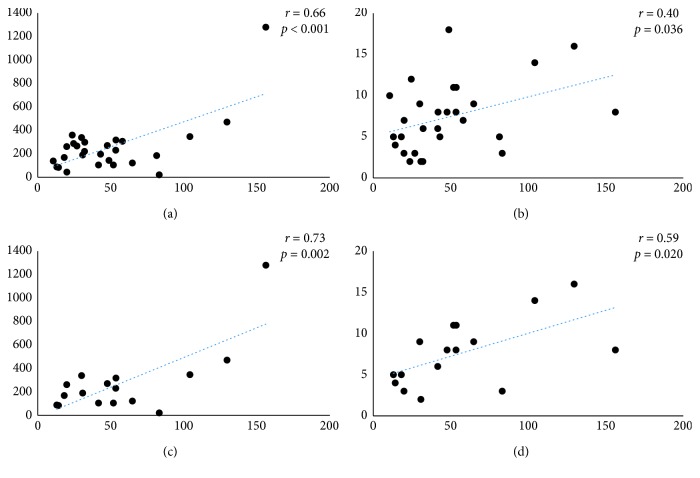
Relationship between the serum hemeoxygenase-1 (HO-1) level and blood biomarker levels. There was a significant correlation between the serum HO-1 and the serum surfactant protein- (SP-) D and the ground glass opacity (GGO) score; however, there was no significant correlation of serum HO-1 with serum KL-6 or with the honeycomb score both in all enrolled patients (a, b) and IPF patients (c, d).

Among all enrolled patients, there was a significant correlation between the serum HO-1 and the serum SP-D (*r*=0.66, *p* < 0.001) and between the serum HO-1 and the GGO score (*r*=0.40, *p*=0.036) (Figures 2(a)and 2(b)); however, there was no significant correlation of the HO-1 level with the serum KL-6 or with the honeycomb score. Also, among patients with IPF, there was a stronger correlation between the serum HO-1 and the serum SP-D (*r*=0.73, *p*=0.002) and between the serum HO-1 and the GGO score (*r*=0.59, *p*=0.020) (Figures 2(c) and 2(d)).

### 3.5. Comparison of Serum HO-1 Level between Nonsurvivors (Hospital Death) and Survivors

**Figure 3:**
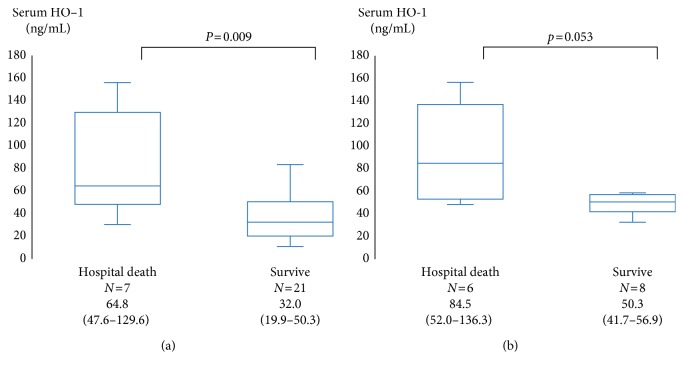
Comparison of the serum hemeoxygenase-1 (HO-1) level between nonsurvivors (hospital death) and survivors. The patients who subsequently died in hospital had presented with significantly higher HO-1 levels than those who survived (64.8 ng/mL vs. 32.0 ng/mL; *p*=0.009) (a). Among patients having an AE, there was the similar tendency between patients who subsequently died in hospital and those who did not die in hospital (84.5 ng/mL vs. 50.3 ng/mL; *p*=0.053) (b). The center bold line is the median value; the bottom and top of the boxes represent the 25^th^ to 75^th^ percentiles, respectively; and the whiskers are 95% confidence intervals.

Seven of 28 patients with IP died in hospital, including 6 patients having an AE and 1 patient not having an AE. The median interval from admission to hospital death was 63 (31–97) days. Patients who subsequently died in hospital had presented with significantly higher HO-1 levels than those who did not die in hospital (64.8 ng/mL vs. 32.0 ng/mL; *p*=0.009) (Figure 3(a)). Among patients having an AE, there was the similar tendency between patients who subsequently died in hospital and those who did not die in hospital (84.5 ng/mL vs. 50.3 ng/mL; *p*=0.053) (Figure 3(b)). The ROC curve analysis for the serum HO-1 level was evaluated to predict hospital death among patients having an AE. The AUC was 0.81, and the best cut-off level was 64.8 ng/mL. Using this cut-off level, serum HO-1 had a sensitivity of 67% and a specificity of 86% for predicting hospital death. An AUC for HO-1 was higher than that obtained for other blood biomarkers and HRCT scores and only significant (Table 3).

### 3.6. Comparison of Alveolar Macrophages HO-1 Level between AE of an IPF Patient and a Stable IPF Patient

Our preliminary histological data showed that alveolar macrophages in the AE of an IPF patient (autopsy case) appeared to possess higher HO-1 expression than did macrophages in a stable IPF patient (surgical lung biopsy) (Supplementary Materials (available here)).

## 4. Discussion

Serum HO-1 has been proposed as a marker for lung disease prognosis. HO-1 generates biliverdin IXα, ferrous iron, and CO from the oxidation of heme, and exhaled CO reflects active heme metabolism [18]. We previously reported that arterial carboxyhemoglobin (CO-Hb, the end-product of heme metabolism) levels are elevated in patients with ILD, particularly during an AE, and these levels have been shown to correlate with parameters that reflect lung inflammation [19]. Therefore, in the present research, we evaluated whether serum HO-1 measurement could be a useful marker for estimating ILD severity and disease prognosis.

Samples from fibrotic ILD patients having an AE exhibit diffuse alveolar damage superimposed on chronic fibrosis when evaluated by histology [14–16]. We found that serum HO-1 levels in the patients having an AE were significantly higher than levels in patients not having an AE and that alveolar macrophages in the AE of an IPF patient appeared to possess higher HO-1 expression than did macrophages in a stable IPF patient. Based on this observation, we hypothesized that the increase in serum HO-1 reflects elevated expression of HO-1 in lung tissue, suggesting that HO-1 might serve as a useful biomarker for diagnosis of AE.

In the presence of alveolitis, the surfactant apoproteins (e.g., SP-D and SP-A) are secreted by type II pneumocytes; these apoproteins can be detected in the serum as a biomarker of alveolitis [20, 21]. Serum SP-D level is reported to be correlated with the extent of alveolitis (denoted by GGO on HRCT), but not with the progression of fibrosis [22]. In the present study, serum HO-1 also significantly correlated with serum SP-D and the GGO score, but not with serum KL-6 or the fibrosis score. Furthermore, unlike serum SP-D, the serum HO-1 level of the patients having an AE was significantly higher than that of the patients not having an AE. Therefore, we speculated that serum HO-1 provides a highly specific marker of alveolitis in patients having an AE, in contrast to serum SP-D, levels of which can be increased due to collagen vascular diseases, congestive heart failure, community-acquired pneumonia, and so on [23–25].

Biomarkers for estimating the disease prognosis of the patients having an AE are not well established in the clinical setting. A previous study reported a composite scoring system for patients with AEs [26]. This scoring system incorporated four individual measurements, including serum LDH (cut-off value, 280 IU/L), serum KL-6 (cut-off value, 1000 IU/L), PaO_2_/FiO_2_ ratio (cut-off value, 100), and extent of abnormal HRCT findings. Using a single serum biomarker, it may be difficult to estimate the disease prognosis of a patient having an AE; however, we found that the patients who subsequently died in hospital had presented with significantly higher HO-1 levels than those who did not die in hospital. This result is consistent with our previous observation, in patients with ARDS, that serum HO-1 levels during intensive care unit stays were persistently higher in individuals who subsequently died (compared to those of survived these incidents) [11]. Therefore, we speculated that serum HO-1 measurement may be useful for predicting hospital mortality among patients having an AE.

## 5. Conclusions

We speculate that serum HO-1 may serve as a useful biomarker for detecting an AE, for evaluating lung inflammation, and for predicting hospital mortality among patients with IP. However, the research described here does include several limitations. First, ours was a single-center study with a small number of patients. This work will need to be expanded to a multicenter prospective study to evaluate the reproducibility of these results. Second, the clinical diagnoses of enrolled patients were heterogeneous, although there was no significant difference between the AE group and the non-AE group. Future research will need to evaluate the clinical utility of serum HO-1 measurement in patients with each of the various histopathological diagnoses (e.g., IPF, NSIP, and organizing pneumonia).

## Figures and Tables

**Table 1:** Patients' characteristics.

Characteristics	Overall patients (A)	Having an acute exacerbation (B)	Not having an acute exacerbation (C)	*p* value (B vs. C)
Total number	28	14	14	
Age (years)	73 (64–79)	76 (66–80)	70 (63–76)	0.131
Male sex, *N* (%)	17 (61)	9 (64)	8 (57)	0.699
Parameters				
Serum hemeoxygenase-1 (ng/mL)	41.7 (23.9–56.9)	53.5 (46.2–88.5)	24.1 (17.1–31.1)	<0.001
Serum lactate dehydrogenase (U/L)	320.0 (224.8–435.5)	370.0 (274.5–463.5)	240.5 (190.5–336.5)	0.017
PaO_2_/FiO_2_ ratio	280.5 (204.1–357.7)	239.0 (178.4–321.2)	341.9 (229.8–398.6)	0.046
Serum surfactant protein-A (ng/mL)	76.8 (55.6–118.0)	75.6 (61.2–124.0)	77.9 (50.6–101.0)	0.325
Serum surfactant protein-D (ng/mL)	221.0 (122.0–307.0)	231 (113.5–332.5)	193.5 (126.3–290.8)	0.544
Serum Krebs von den Lungen-6 (U/mL)	1005.0 (616.0–2152.0)	1056 (720.0–2454.5)	977.0 (354.8–2049.3)	0.482
Ground glass opacity score	7.0 (4.3–9.8)	8.0 (6.8–11.8)	5.0 (3.0–7.5)	0.024
Honeycomb score	6.0 (0.3–8.8)	8.0 (3.0–8.3)	1.0 (0.0–9.5)	0.156
Diagnosis, *N* (%)				
Idiopathic pulmonary fibrosis	16 (57)	10 (71)	6 (43)	0.127
Nonidiopathic pulmonary fibrosis	12 (43)	4 (29)	8 (57)	0.127
Outcome				
Hospital death, *N* (%)	7 (25)	6 (43)	1 (7)	0.023

Values are reported as median with 25–75 percentiles or %, unless otherwise indicated.

**Table 2:** The receiver operating characteristic curve for the serum HO-1 level (detecting an AE patient).

Variable	Area under the ROC curve	Best cut-off values	Sensitivity (%)	Specificity (%)	*p* value
Serum hemeoxygenase-1 (ng/mL)	0.93	41.6	93	86	0.010
Serum lactate dehydrogenase (U/L)	0.77	255	93	64	0.034
PaO_2_/FiO_2_ ratio	0.73	302	77	62	0.036
Serum surfactant protein-A (ng/mL)	0.63	55.6	100	45	0.595
Serum surfactant protein-D (ng/mL)	0.57	307	38	46	0.249
Serum KL-6 (U/mL)	0.58	616	92	36	0.700
Ground glass opacity score	0.75	7	79	71	0.036
Honeycomb score	0.66	1	100	50	0.233

**Table 3:** The receiver operating characteristic curve for the serum HO-1 level (predicting hospital death among patients having an AE).

Variable	Area under the ROC curve	Best cut-off values	Sensitivity (%)	Specificity (%)	*p* value
Serum hemeoxygenase-1 (ng/mL)	0.81	64.8	67	86	0.025
Serum lactate dehydrogenase (U/L)	0.57	273	100	29	0.806
PaO_2_/FiO_2_ ratio	0.58	340	100	33	0.506
Serum surfactant protein-A (ng/mL)	0.57	116	60	83	0.550
Serum surfactant protein-D (ng/mL)	0.81	231	83	67	0.055
Serum KL-6 (U/mL)	0.56	1821	83	50	0.617
Ground glass opacity score	0.77	8	100	57	0.062
Honeycomb score	0.56	7	50	71	0.912

## Data Availability

The table and figure data used to support the findings of this study are included within the article.

## Abbreviations

AE: Acute exacerbation
ARDS: Acute respiratory distress syndrome
AUC: Area under the ROC curve
CO-Hb: Carboxyhemoglobin
CT-ILD: Connective tissue disease-associated ILD
ELISA: Enzyme-linked immunosorbent assay
FiO_2_: Fraction of inspiratory oxygen
GGO: Ground glass opacity
HO-1: Hemeoxygenase-1
HRCT: High-resolution CT
ICU: Intensive care unit
ILD: Interstitial lung disease
iNSIP: Idiopathic nonspecific interstitial pneumonia
IP: Interstitial pneumonia
IPF: Idiopathic pulmonary fibrosis
KL-6: Krebs von den Lungen-6
LDH: Lactate dehydrogenase
PaO_2_: Partial pressure of oxygen in arterial blood
ROC: Receiver operating characteristics
SP: Surfactant protein.

## Supplementary Materials

Supplementary Materials Immunohistochemical staining for hemeoxygenase-1 (HO-1) expression in lung specimens (A). Bacterial pneumonia (B). Acute exacerbation (AE) of idiopathic pulmonary fibrosis (IPF) (C). Stable IPF in an AE-IPF patient (autopsy case); high expression of HO-1 was observed mainly in alveolar macrophages, while expression of HO-1 in fibrotic lesions/alveolar macrophages was not conspicuous in stable IPF (the specimen of surgical lung biopsy) (original magnification × 200).
