# Supplementary figures and images for: Clinical Significance of Serum Hemeoxygenase-1 as a New Biomarker for the Patients with Interstitial Pneumonia
Source: Can Respir J. 2018 Nov 22;2018:7260178. doi: 10.1155/2018/7260178 (PMC6282126; doi:10.1155/2018/7260178)

## Slide 1
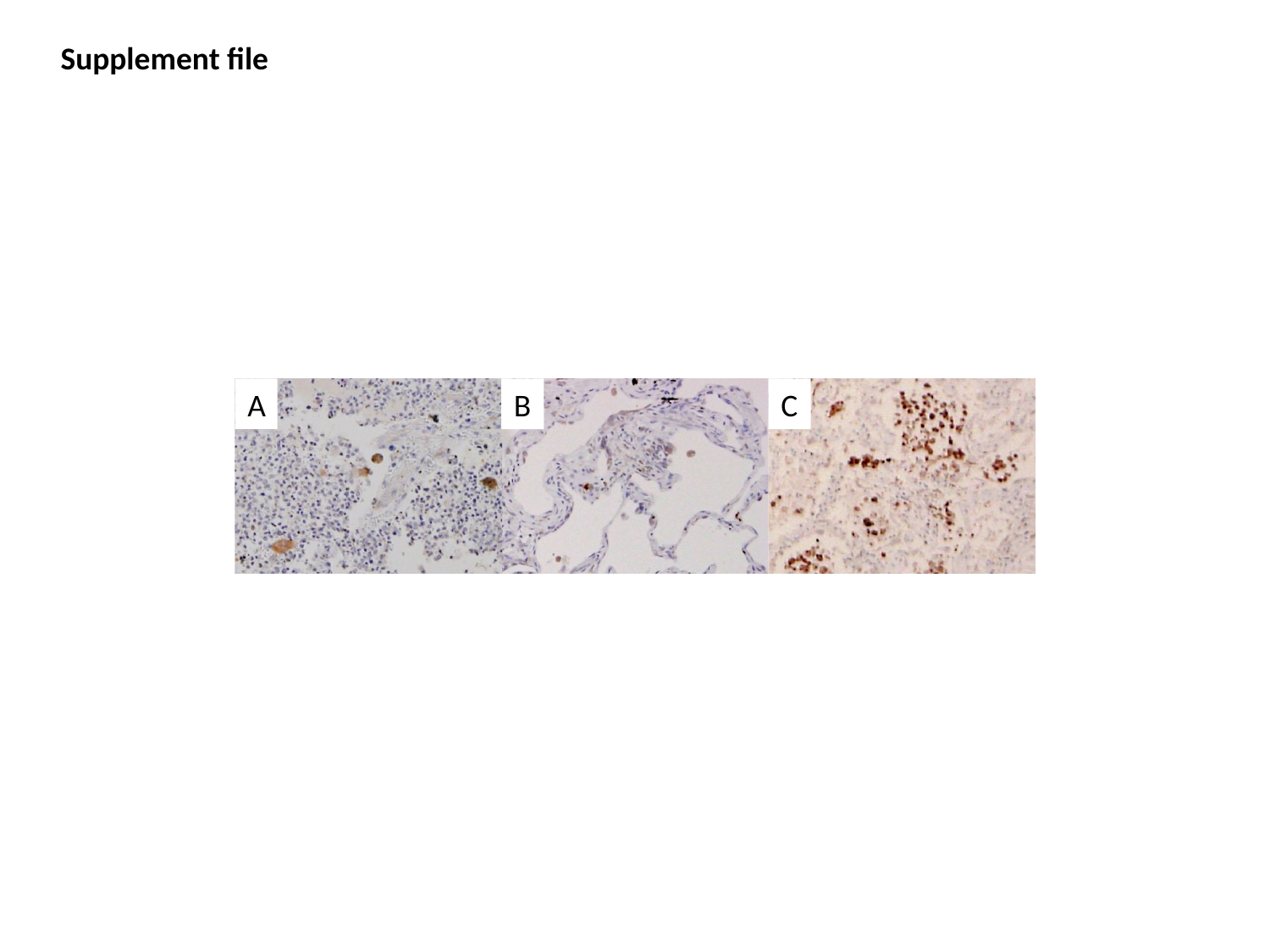

Supplement file
A
B
C

Supplement: Supplementary Materials — Immunohistochemical staining for hemeoxygenase-1 (HO-1) expression in lung specimens (A). Bacterial pneumonia (B). Acute exacerbation (AE) of idiopathic pulmonary fibrosis (IPF) (C). Stable IPF in an AE-IPF patient (autopsy case); high expression of HO-1 was observed mainly in alveolar macrophages, while expression of HO-1 in fibrotic lesions/alveolar macrophages was not conspicuous in stable IPF (the specimen of surgical lung biopsy) (original magnification × 200). [file 7260178.f1.pptx]
